# Bilateral-pectoral major muscle advancement flap combined with vacuum-assisted closure therapy for the treatment of deep sternal wound infections after cardiac surgery

**DOI:** 10.1186/s13019-020-01264-2

**Published:** 2020-08-27

**Authors:** Feng Song, Zhenzhong Liu

**Affiliations:** The Department of Plastic Surgery, The Second Hospital, Cheeloo College of Medicine, Shandong University, No. 247 Beiyuan Street, Jinan, 250000 Shandong Province China

**Keywords:** Cardiac surgery, Deep sternal wound infections, Bilateral-pectoral major muscle advancement flap, Vacuum-assisted closure therapy

## Abstract

**Objectives:**

The median sternotomy is the most common surgical approach for cardiac surgery. Deep sternal wound infection is a fatal complication after median sternotomy. The aim of this study was to evaluate the therapeutic effect of Bilateral-pectoral major muscle advancement flap combined with Vacuum-assisted closure therapy on rehabilitation for the treatment of deep sternal wound infection after cardiac surgery.

**Methods:**

Between January 2016 to January 2018, 21 patients (10 males, 11 females) with deep sternal wound infection after cardiac surgery underwent Bilateral-pectoral major muscle advancement flap combined with Vacuum-assisted closure therapy. These patients were followed-up 12 months postoperative. The patient characteristics, duration of vacuum-assisted closure therapy, the mean hospital stay, postoperative complications, long-term survival of patients were retrospectively analyzed.

**Results:**

Most patients undergone 1–3 times vacuum-assisted closure treatment sessions before closure. All patients were cured to discharge, the mean hospital stay was 21.1 days. Most patients’ healing wounds were first-stage healing, only one patient’s wound was second-stage healing, none was third-stage healing. One patient developed pulmonary infection and respiratory failure during the 12-month follow-up. None of the patients died during follow-up.

**Conclusions:**

Bilateral-pectoral major muscle advancement flap combined with Vacuum-assisted closure therapy for the treatment of deep sternal wound infections after cardiac surgery can shorten the hospital stays and few complications. However, this is a retrospective case series presentation with no comparison group, the number of inferences is limited, so further large-scale controlled studies are needed.

## Introductions

The median sternotomy is the most common surgical approach for cardiac surgery. Once the median sternotomy is infected, it eventually develops into deep sternal wound infection (DSWI). DSWI is defined as infection involving fascia or deeper with at least one of the following: evidence of infection seen at re-operation or spontaneous dehiscence, positive culture of mediastinal fluid and/or positive blood culture and/or chest pain with sternal instability and temperature higher than 38 degrees Celsius’ [1]. DSWI is a significant complication which occurs in 0.8–8% of patients after median sternotomy [2], and the mortality rates from 8 to 45% [3–5]. A pedicled omental flap has been reported for the treatment of DSWI, but many complications were prone to occur, which can lead to the failure of treatment [6]. Vertical and transversal rectus abdominis muscle flap has also been reported, but they can only be used for DSWI at the lower edge of the sternum [7]. At present, the pectoralis major muscle transfer flap (PMMTF) is the main method [8, 9]. But this method needs to cut off the lateral muscle fibers of the pectoralis major muscle which affect the function of the upper limbs and damage the important source of blood supply of the pectoralis major.

Vacuum-assisted closure (VAC) therapy has been widely used for the treatment of wound infection. VAC can improve healing of DSWI by increasing wound blood flow, reducing bacterial loads, enhancing formation of granulation tissue [10]. VAC therapy has shown promising results in the treatment of DSWI after cadiac surgery comparison with other therapeutic options. And it has no standardized procedure, various strategies are being used. The basic principle of operation is debridement, administration of culture-specific antibiotics, wound closure therapy.

In this review, we present our experience in treating DSWI after cardiac surgery with Bilateral-pectoral major muscle advancement flap combined with Vacuum-assisted closure therapy and the results were satisfactory.

## Materials an methods

### Clinical data

The study was approved by the hospital ethics committee and informed consent of patients. Twenty-one patients with DSWI after cardiac surgery admitted to the Department of Plastic Surgery, Second Hospital of Shandong University from January 01, 2016 to January 01, 2018. They all received the treatment of Bilateral-pectoral major muscle advancement flap combined with VAC. These patients were followed-up 12 months at least postoperative. Patients included criteria: 1. Patients meet the diagnostic criteria for DSWI. 2. Patients approved by the Ethics Committee. Exclusion criteria: Patients who have basic diseases are not suitable for surgery. The patient characteristics, duration of vacuum-assisted closure therapy, the mean hospital stay, postoperative complications, long-term survival of patients were retrospectively analyzed.

### DSWI definition

According to the US Centre for Disease Control and Prevention, DSWI is defined as Infection involving fascia or deeper with at least one of the following: evidence of infection seen at re-operation or spontaneous dehiscence, positive culture of mediastinal fluid and/or positive blood culture and/or chest pain with sternal instability and temperature higher than 38 degrees Celsius [1].

### Surgical technique

**Fig. 1:**
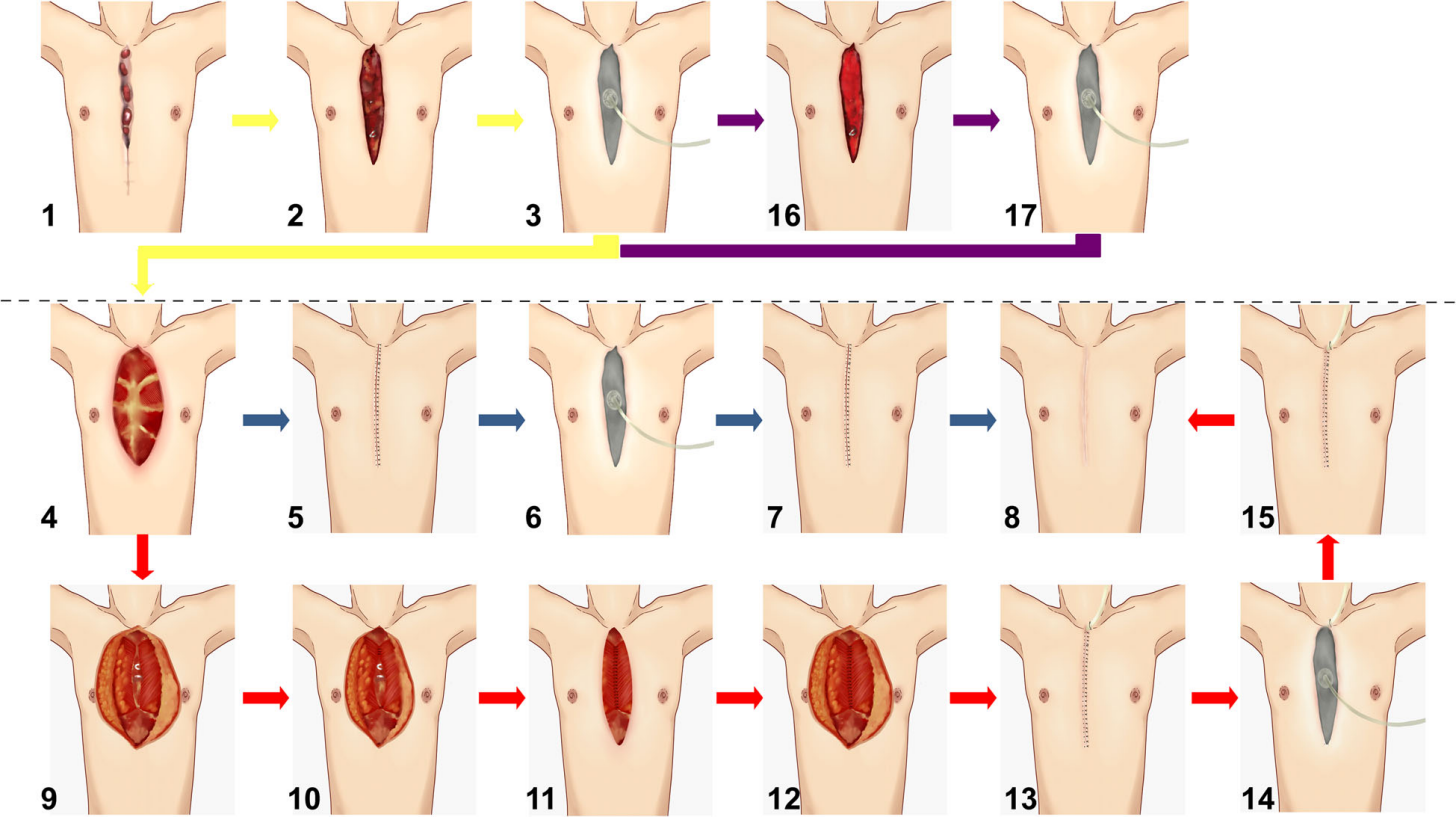
Methods and Surgical Technique. 1 DSWI after cardiac surgery; 2. The wound after debridement; 3. Using VAC after debridement; 4. The wound after debridemen and VAC; 5. The skin and subcutaneous tissue are sutured by methods of relieving tension; 6. Using VAC after retension suture of the skin; 7. VAC is removed; 8. Wound healing; 9. Separate the starting points of the bilateral pectoralis major muscles; 10. Advance the bilateral pectoralis major muscles to the middle; 11. Suture the bilateral pectoralis major muscles; 12. Reduce the tension of the flaps; 13. The skin and subcutaneous tissue is sutured by methods of relieving tension; 14. Using VAC after retension suture of the skin; 15. VAC is removed; 16.The wound after second debridement; 17. Using VAC after second debridement

All patients with DSWI are well prepared preoperatively to have better tolerance to surgery. Such as appropriate blood pressure, appropriate blood sugar, hemoglobin content, albumin content, cardiopulmonary function. (1) Anesthesia. All patients are anesthetized with tracheal intubation. Patients with poor cardiac function plus local anesthesia; (2) Thorough debridement. All nonviable bone and staples are removed. Use a rongeur and curette to scrap the diseased sternum to reach normal bone tissue. The end point of debridement is marked by the appearance of bleeding in the cortical bone. Then it is necessary to explore the presence or absence of infection in the posterior sternum. If there is an infection in the mediastinum, the infected tissue is scraped off until fresh bleeding. If there is infected rib at the same time, the infected rib is removed. The standard for successful resection is to reach normal bone tissue. It continues with a copious irrigation of the wound with the solution containing hydrogen peroxide and normal saline. Internal fixations that do not affect the stability of the sternum are removed, such as ligating the wire. The internal fixation that affects the stability of the sternum is temporarily reserved for secondary surgery. After 1 week, it is decided whether to debride again according to the condition of the wound. (3) VAC Using the negative pressure device of Cool Levi, the drainage tube and the film are cut to the appropriate size according to the size of the wound. Connected to the vacuum device, the negative pressure is -75 mmHg to -100 mmHg. The mode is intermittent vacuum suction; (4) Postoperative treatment:supportive treatment. According to the results of bacterial culture or drug susceptibility test of wound secretion, sensitive antibiotics are selected to prevent infection during perioperative period. (5) Second-stage surgery is performed after 1 week of debridement. VAC is removed. If the sternum is unstable, the internal fixation may be retained. If debridement is not thorough, it needs to be debrided again. After second debridement, VAC is used again. In short, debridement must be thorough. The use of BPMMAF is determined according to the size of the wound. If do not need to use BPMMAF, skin and subcutaneous tissue are sutured by methods of relieving tension and VAC is used again. VAC and the suture are removed 10–12 days after surgery. If the wound cavity is large, the use of BPMMAF is necessary. Cut off at the beginning of the pectoralis major (sternum and part of the ribs) and separate the pectoralis major muscles. BPMMAF is advanced toward the midline site and then sutured by methods of relieving tension intermittently. The purpose is to fill the sternal defect and pressurize the sternum. The subcutaneous tissue and pectoralis major muscles are correctly separated, and the skin is sutured in layers. The drainage tube is placed under the muscle flap. According to the level of anatomy, the skin is suture with reduced-tension. After closing the chest wound, VAC is used at the incision site again. The drainage tube is pulled out 3–5 days after surgery based on the amount of drained fluid. VAC and the suture are removed 10–12 days after surgery. (Fig. 1).

#### Case 1

Methods and surgical technique of VAC in patient. The patient’s condition only requires VAC as the treatment. (Figure 1)

#### Case 2

**Fig. 2:**
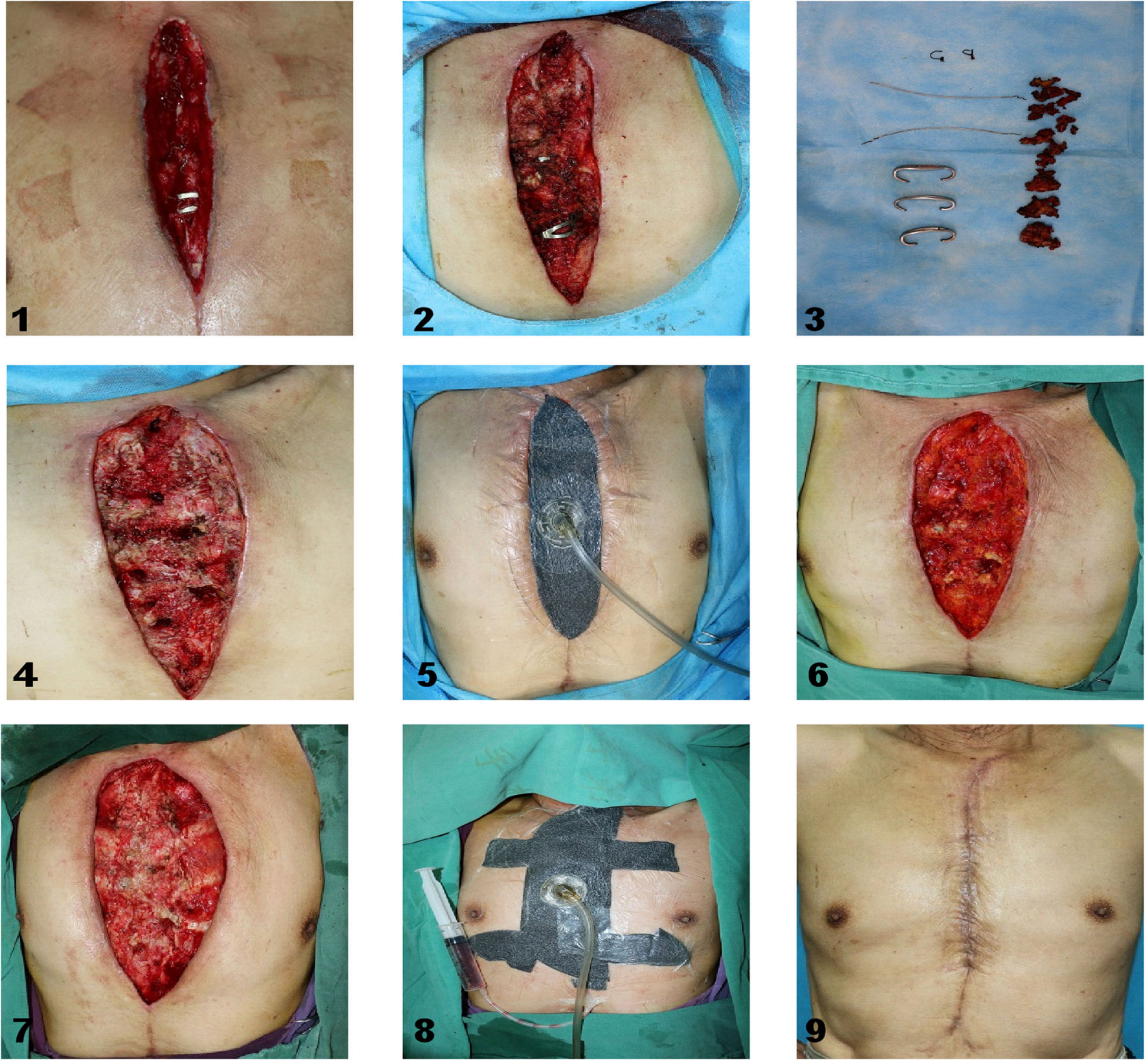
Long-term result and surgical technique of VAC in patient. 1. Chronic osteomyelitis wound after cardiac surgery; 2. Enlarged chronic osteomyelitis wound; 3. Cleared necrotic tissue, knot, wire and diseased sternum; 4. The wound after debridement; 5. Using VAC after debridement; 6. The wound after removing VAC; 7. Reduce the tension of the flaps on both sides; 8. VAC after retension suture of the skin; 9. Wound healing

Methods and surgical technique of bilateral-pectoral major muscle advancement flap combined with VAC in patient. The patient’s condition must require bilateral-pectoral major muscle advancement flap and VAC as the treatment. (Fig. 2).

#### Case 3

**Fig. 3:**
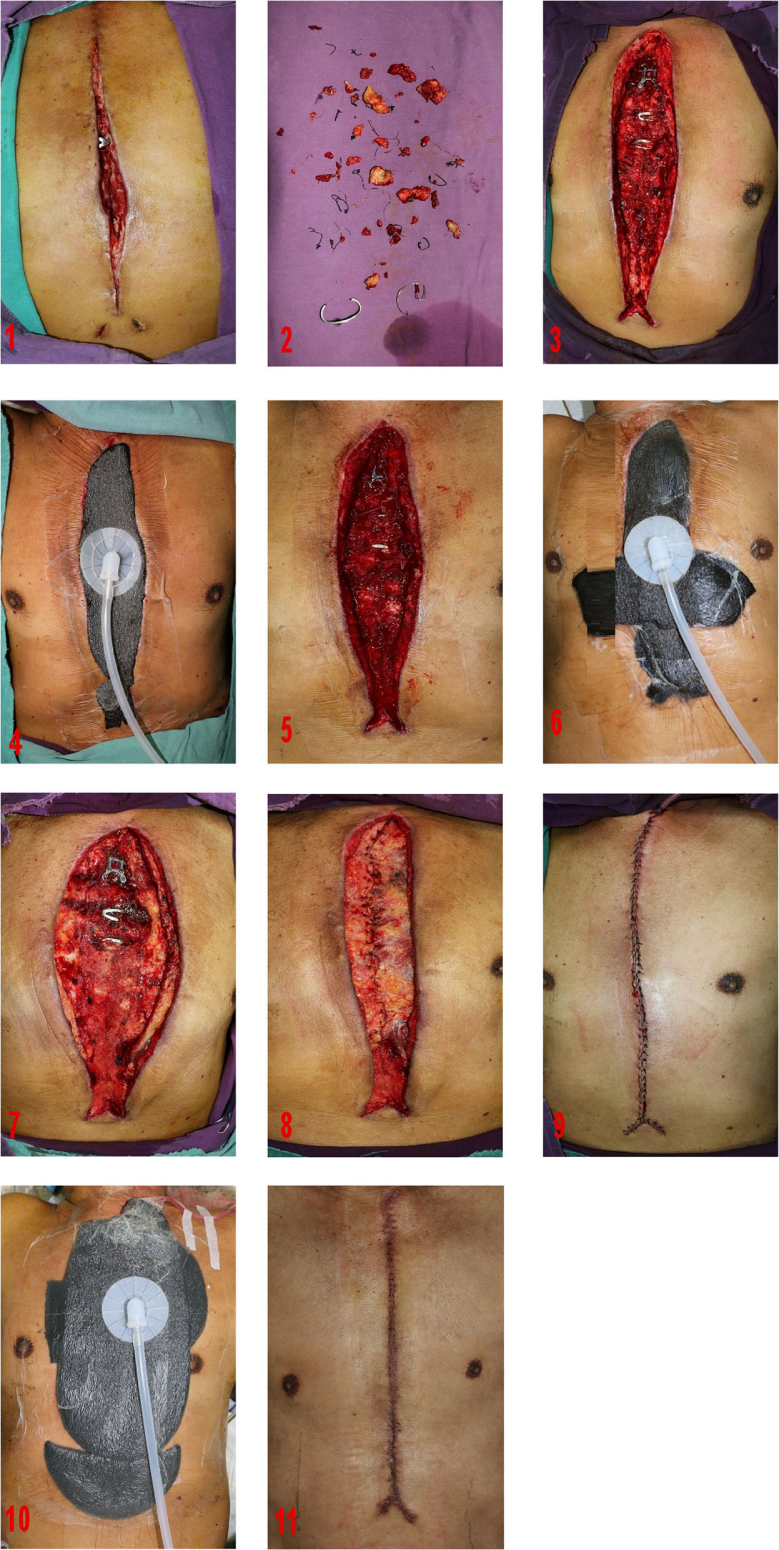
Long-term result and surgical technique of BPMMAF combined with VAC in patient.1 .DSWI after cardiac surgery; 2. Cleared necrotic tissue, knot, wire and diseased sternum; 3.The wound after debridement; 4. Using VAC after debridement; 5. The wound after second debridement; 6. Using VAC after second debridement; 7. Separate the starting points of the bilateral pectoralis major muscles; 8. Advance the bilateral pectoralis major muscles to the middle; and suture the bilateral pectoralis major muscles; 9.The skin and subcutaneous tissue is sutured by methods of relieving tension; 10. Using VAC after retension suture of the skin; 11.Wound healing

The condition of the wound needs second or more debridement. In short, debridement must be thorough. (Fig. 3).

## Results

Patients all received the treatment of Bilateral-pectoral major muscle advancement flap combined with VAC. There were 10 males (47.6%) and 11 females (52.4%) with an average age of 58.1 years (range 45 to 78 years). Patients all underwent median sternotomy, 16 patients (76.2%) for coronary artery bypass procedures (CABG), 5 patients (23.8%) for cardiac valve replacement (VALVE) (Table 1). These patients were followed-up 12 months at least postoperative.

**Table 1:** Clinical dataTable1 Clinical data

***Preoperative characteristics***	
Male-Female Ratio	10:11
Age (years): average and range	58.1 (45–78)
***Surgical procedure***	Number (%)
Coronary artery bypass graft (CABG)	16 (76.2%)
Valve replacement (VALVE)	5 (23.8%)

The average time interval between primary sternotomy and clinical manifestations of DSWI was 20.7 days, most patients (9 of 21, 42.8%) had clinical manifestations from 8 to 14 days postoperative. Parts patients (7 of 21, 52.3%) underwent debridement from 15 to 28 days were detected, 3 patients (14.3%) were detected within 14 days after infection, 2 patient (9.5%) were detected more than 29 days (Table 2).

**Table 2:** Time interval between primary sternotomy and clinical manifestations of DSWI

	DSWI	
Time interval	Number (%)	Mean
≤7 days	3 (14.3)	20.7 days
8–14 days	9 (42.8)	
15–28 days	7 (33.3)	
≥29 days	2 (9.5)	
Total	21	

Wound cultures were positive in 18 patients (85.7%), the most common pathogens were Gram-positive cocci (GPC) and Gram-negative bacilli (GNB), such as *Pseudomonas aeruginosa* (14.3%), *Acinetobacter baumannii* (14.3%), *Enterobacter cloacae* (4.8%), *Staphylococcus aureus* (23.8%), *Staphylococcus epidermidis* (14.3%), and *Enterococcus faecalis* (4.8%). (Table 3).

**Table 3:** Pathogenic data of the 18 cases of DSWI

Etiology	Patients with a diagnosis of etiology [n (%)]
GNB	7 (33.3)
*Pseudomonas aeruginosa*	3 (14.3)
*Acinetobacter baumannii*	3 (14.3)
*Enterobacter cloacae*	1 (4.8)
GPC	9 (42.8)
*Staphylococcus aureus*	5 (23.8)
*Staphylococcus epidermidis*	3 (14.3)
*Enterococcus faecalis*	1 (4.8)
Fungi	1 (4.8)
Mixed infections	1 (4.8)

Among these 21 patients, debridement and VAC was performed 1–3 times before the DSWI was rehabilitate, of which 5 patients (27.3%) underwent once, 14 patients (66.7%) underwent twice (Fig. 2) and 3 patients (14.3%) underwent three times (Fig. 3). (Table 4).

**Table 4:** Number of debridement operations

Number of debridement	Number (%)
1	5 (23.8)
2	14 (66.7)
3	3 (14.3)
Total	21

Twenty patients (95.2%) were healed in the first stage, one patient (4.8%) was healed in the second stage, and none was healed in the third stage. Only 15 patients had the testing of pectoralis major muscle strength and pulmonary function after surgery. Among 15 patients, the movement of the upper limbs is normal, and there is no obvious abnormal respiratory function (Table 5). One patient developed pulmonary infection and respiratory failure during the 12-month follow-up. None of the patients died during follow-up.

**Table 5:** Comparison of Preoperative and Postoperative Series

		Preoperative Condition (*n* = 21)	Postoperative Condition (*n* = 15)	*P* value
Strength test of	Left	V	V	–
Pectoralis Major muscle	Right	V	V	–
Pulmonary function	Vital	3022 ± 95.51	3103 ± 106.7	0.5824
testing	Capacity (ml)			

The mean hospital stay from debridement was21.1 days, 6 patients (28.6%) were less than or equal to 14 days, from 15 to 28 days in 13 patients (61.9%), more than 29 days in 2 patients (9.5%) (Table 6).

**Table 6:** Hospital stays from surgical debridement

Swz	Number (%)	Mean
≤14 days	6 (28.6)	21.1 days
15–28 days	13 (61.9)	
≥29 days	2 (9.5)	
Total	21	

## Discussion

The median sternotomy is the most common surgical approach for cardiac surgery. It is widely used because of its sufficient exposure and convenient operation. However, once the median sternotomy is infected, especially the deep incision infection, treatment is very difficult. These increase suffering of patient, prolong hospital stays, and increase treatment costs. It is difficult to cure after debridement treatment, and the incision infection that has not healed eventually develops into DSWI. The incidence of this disease is between 0.4 and 5.0%. There are many causes of deep sternal incision infection [11, 12], such as diabetes, obesity, advanced age, chronic obstructive pulmonary disease, long-term heavy smoking, chronic renal failure, immune function, low heart function, long operation time, excessive bleeding, and malnutrition.

At present, the main treatment of DSWI is the application of tissue flap transfer after debridement. After a lot of clinical experience, the pectoralis major muscle flap (PMMF) has great advantages. The main advantages of the PMMF: (1) The pectoralis major muscle is close to the sternum, easy to dissociate, no need for another incision. The flap size can be adjusted according to the size of the wound; (2) The blood supply of the pectoralis major muscle is rich, such as the intercostal artery. Therefore, the PMMF is the main treatment for tissue flap repair now. Among them, PMMTF is the main method. However, this method requires cutting off the lateral muscle fibers of the pectoralis major, which affects the function of the upper limbs. This method needs to cut or damage the intercostal perforating vessels and the thoracodorsal peak arteries, which will affect the blood supply of the muscle flaps, because the perforating vessels and the thoracic and thoracic peak arteries are important blood supply sources for the pectoralis major.

The bilateral-pectoral major muscle advancement flap was used in our team to treat chronic sternal osteomyelitis. By the method, the muscle fibers on the outer side of the pectoralis major muscle can be retained, which preserves the function of the pectoralis major muscle and thus does not affect the function of the upper limbs. During the operation, only the starting position of the pectoralis major muscle is changed, and the upper limbs function is not affected at all. According to the size and location of the defect, the trauma can be reduced and the time of operation can be shortened. Because the starting point of the pectoralis major muscle (the sternum and part of the rib) is cut off, the bilateral pectoralis major muscle flap is advanced to the midline, which can well protect the intercostal artery perforator and the thoracic and shoulder arteries to prevent muscle ischemia.

The theory of wet healing is a new concept in recent years, which can provide a better outcome, less pain and less scar formation. VAC can provide a moist environment for wounds, which is a great improvement in wound healing treatment. The treatment of VAC not only promotes vascular proliferation, but also promotes differentiation of cells around the new capillary. For soft tissue defects and dead space, VAC can gradually achieve the purpose of eliminating dead space. Continuous attraction should be avoided, and continued attraction can lead to poor local blood circulation and chronic ischemia. Intermittent attraction can cause reactive hyperemia and increase blood flow into the wound. When the negative pressure is stopped, the poorly perfused tissue is reactively hyperemic, increasing the oxygen and nutrients of the wound and increasing the waste removal. In short, the main reasons for VAC can promote local blood flow, reduce bacterial growth, increase the growth rate of wound granulation tissue, shrink wounds, and promote epithelial growth.

To summarize the experience of using bilateral-pectoral major muscle advancement flap combined with VAC for DSWI. The following points are necessary: (1) Thorough debridement. Remove the suture, dead bone, necrotic tissue and granulation tissue in the lesion. It is important to eliminate the source of infection completely, and soak with hydrogen peroxide and iodophor solution to make the surgical area relatively sterile. After debridement, DSWI is used; (2) DSWI is rationally used. DSWI can fill the incision, pressure the sternum and reduce the tension of the flap. (3) Apply the appropriate size of the PMMF to fill the wound. Do not leave a dead space to prevent re-infection. (4) Internal fixation that does not affect the stability of the sternum is removed, such as ligation wire. The internal fixture that affects the stability of the sternum is temporarily reserved for secondary surgery. According to the stability of the sternum, the second surgery determines whether the fixture is removed. The stability of the sternum after VAC is generally increased. The loosened wire is fixed, and the wire and sternum claws which have no fixed function are removed. (5) Bilateral pectoralis major muscles and wounds should completely stop bleeding. There is no blood and effusion in the wound to prevent re-infection.

This study demonstrates that bilateral-pectoral major muscle advancement flap combined with VAC is effective in the treatment of DSWI. The recurrence rate is low, the operation procedure is simple, and there is no need to increase the surgical incision. Patients are more likely to undergo this type of surgery. It is easier for clinicians to master. Satisfactory long-term results, and almost no complications.

## Conclusion

Bilateral-pectoral major muscle advancement flap combined with Vacuum-assisted closure therapy for the treatment of deep sternal wound infections after cardiac surgery can shorten the hospital stays and few complications. However, this is a retrospective case series presentation with no comparison group, the number of inferences is limited, so further large-scale controlled studies are needed.

## Data Availability

Not applicable.

## Abbreviations

CABG: Coronary artery bypass procedures
DSWI: Deep sternal wound infection
GPC: Gram-positive cocci
GNB: Gram-negative bacilli
PMMF: Pectoralis major muscle flap
PMMTF: Pectoralis major muscle transfer flap
VAC: Vacuum-assisted closure
VALVE: Cardiac valve replacement
